# Normal FGF-21-Serum Levels in Patients with Carnitine Palmitoyltransferase II (CPT II) Deficiency

**DOI:** 10.3390/ijms20061400

**Published:** 2019-03-20

**Authors:** Leila Motlagh Scholle, Diana Lehmann, Pushpa Raj Joshi, Stephan Zierz

**Affiliations:** Department of Neurology, Martin-Luther-University Halle-Wittenberg, Ernst-Grube-Str. 40, 06120 Halle (Saale), Germany; diana.lehmann@rku.de (D.L.); pushpa.joshi@medizin.uni-halle.de (P.R.J.); stephan.zierz@uk-halle.de (S.Z.)

**Keywords:** FGF-21, mitochondrial diseases, carnitine palmitoyltransferase II deficiency, biomarker

## Abstract

Fibroblast growth factor 21 (FGF-21) is known to be a biomarker for mitochondrial disorders. An upregulation of FGF-21 in serum and muscle of carnitine palmitoyltransferase I (CPT I) and carnitine palmitoyltransferase II (CPT II) knock-out mice has been reported. In human CPT II deficiency, enzyme activity and protein content are normal, but the enzyme is abnormally regulated by malonyl-CoA and is abnormally thermolabile. Citrate synthase (CS) activity is increased in patients with CPT II deficiency. This may indicate a compensatory response to an impaired function of CPT II. In this study, FGF-21 serum levels in patients with CPT II deficiency during attack free intervals and in healthy controls were measured by enzyme linked immunosorbent assay (ELISA). The data showed no significant difference between FGF-21 concentration in the serum of patients with CPT II deficiency and that in the healthy controls. The results of the present work support the hypothesis that in muscle CPT II deficiency, in contrast to the mouse knockout model, mitochondrial fatty acid utilization is not persistently reduced. Thus, FGF-21 does not seem to be a useful biomarker in the diagnosis of CPT II deficiency.

## 1. Introduction

FGF-21 was first introduced in 2011 as a biomarker for the diagnosis of mitochondrial diseases [1]. This idea has subsequently been confirmed in various other studies [2,3,4,5,6]. The increased expression of FGF-21 in mitochondrial disorders is believed to be a compensatory response to respiratory chain deficiency [7]. In some metabolic diseases other than mitochondrial disorders, like in patients with obesity and type 2 diabetes, the serum or plasma levels of FGF-21 have been reported to be elevated [8].

The β-oxidation of activated fatty acids occurs in the mitochondrial matrix [9,10]. For transport of long chain fatty acids through the mitochondrial inner membrane, a special transport system is needed, including CPT I and CPT II. CPT II deficiency is regarded as the most common defect of lipid metabolism in skeletal muscle. CPT II is a ubiquitous protein without tissue specificity [10,11].

FGF-21 knockout mice (Fgf21^−/−^) have been shown to not be able to mobilize and utilize lipids out of a ketogenic diet [12]. The Fgf21^−/−^ mice showed significantly increased levels of FGF-21 in serum in both fed and fasted states. The mRNA expression of FGF-21 was upregulated in several muscles of these CPT I deficient mice [13]. 

Mice with liver-specific knockout of CPT II (Cpt2^L−/−^) have been shown to have elevated mRNA expressions of *gdf15* and *fgf21* genes and increased serum FGF-21 and Growth/Differentiation factor (GDF)-15 concentrations following high-fat-feeding [14,15]. Another group of mice with an adipose-specific knockout of CPT II (CPT2^A−/−^) [16] was shown to have an increased expression of mRNA of *fgf21* in brown adipose tissue (BAT) following cold exposure [17]. 

The human muscle form of CPT II-deficiency is characterized by attacks of myalgia and myoglobinura provoked by prolonged exercise, fasting, fever, or exposure to cold [11,18]. In about 90% of patients a p.S113L mutation can be found with an allele frequency of 60–70% [19]. More than 60 other mutations have been identified in patients with CPT II- deficiency [20].

Until now, there have been no studies performed on FGF-21 serum levels in CPT II deficient patients. Our data showed that in contrast to the knockout mouse model, FGF-21 serum levels in patients with CPT II deficiency were not different from those in healthy controls.

## 2. Results

**Figure 1:**
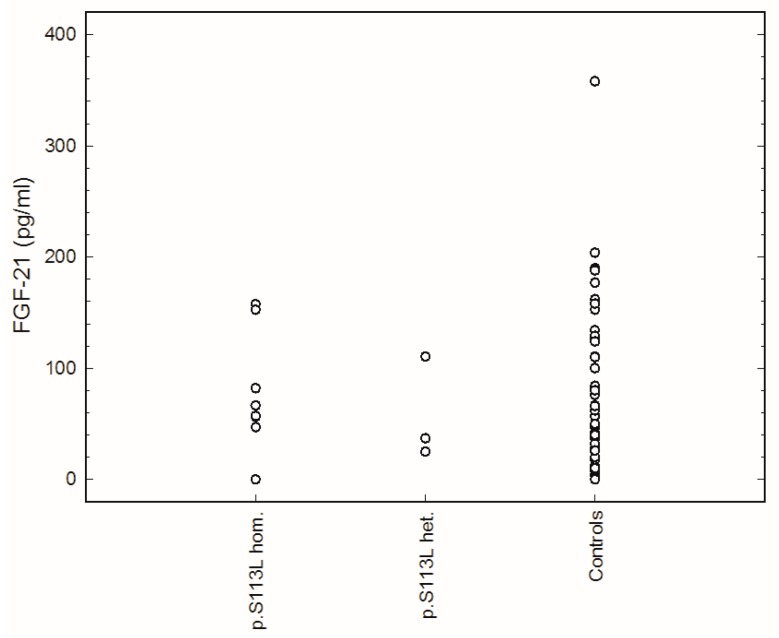
Fibroblast growth factor 21 (FGF-21) serum concentration in controls and patients with carnitine palmitoyltransferase II (CPT II) deficiency (p.S113L hom.: patients homozygous for the common p.S113L mutation; p.S113L het.: Patients with p.S113L on one allele and another mutation on other allele. The detailed genotype of all patients is illustrated in Table 1).

The cut-off concentration of FGF-21 for normal controls was set at 190 pg/mL based on the results of the 95th percentile of controls. None of the 13 CPT II patients had elevated FGF-21 serum concentrations (Table 1). For the whole group of patients, FGF-21 serum concentrations were not significantly different from those of the controls. None of the patients showed an FGF-21 concentration above the cut-off value (Figure 1). Spearman rank correlation coefficient testing showed no significant correlation between FGF-21 concentration and body mass index (BMI) (Spearman correlation (rs = 0.46, *p* = 0.11). There was also no significant correlation between the FGF-21 concentration and the frequency of attacks within one year (rs = 0.15, *p* = 0.62).

## 3. Discussion

In mouse models with liver- and adipose-specific knockout of CPT II (Cpt2^L−/−^) (CPT2^A−/−^), an increase in mRNA expression of Fgf21 genes has been found following high-fat-feeding or exposure to cold [15,16,17]. A significant increase in mtDNA copy number after treatment of C2C12 myoblasts with FGF-21 has been reported. Additionally, the expression of CPT1A, CPT II genes, and citrate synthase activity also increased (1.5 times) [7]. These data suggest an adopting role of FGF-21 in response to a loss of mitochondrial fatty acid oxidation, which has been interpreted as a compensatory process in order to maintain the energy supply in affected tissues [13,15]. 

Based on these findings, a higher FGF-21 serum level could also be expected in CPT II deficient patients. However, in the present study no significant difference was found in FGF-21 serum concentration between CPT II deficient patients and the controls. This outcome can be explained by the following differences between the knock out models and human muscle CPT II deficiency: (i) in contrast to knockout models, there is no loss of CPT II activity in patients [19,21] and (ii) unlike in knockout models there is no reduction of protein concentrations of CPT I and CPT II in patients [22]. The genotypes of the patients are typical for human muscle deficiency. In the mouse model, however, the whole gene is knocked out. Thus, the genotypes are not comparable. 

The increased citrate synthase activity in the muscles of patients might indicate the increased mitochondrial compartment compensating for the functional impairment in CPT II deficiency [22,23,24]. 

It has been suggested that the increase in FGF-21 concentrations in serum might occur with increasing clinical severity, progression of mitochondrial disease, and muscle pathology [1,3,4].

The human muscle form of CPT II deficiency is characterized by attacks of myalgia and myoglobinura, provoked by prolonged exercise, fasting, fever, or exposure to cold [11,18]. 

One possible limitation of our study might be the small number of 13 patients. However, human muscle CPT II deficiency is a rare disease with very low prevalence. Thus the number of 13 patients included in our study is exceptionally high compared to other biochemical and molecular studies on CPT II deficiencies.

It should be noted that the serum samples from our patients were collected during attack-free intervals. Hence, the only attack-like impairment of fatty acid utilization in patients with the muscle form of CPT II deficiency might not have been sufficient to cause an increased FGF-21, at least during attack-free intervals. This is consistent with the notion that in human muscle CPT II deficiency there is no permanent lack of the enzymatic active enzyme but rather an abnormal regulation and thermoinstability of the mutant enzyme [25,26].

## 4. Materials and Methods 

### 4.1. Patients and Controls

Thirteen patients with a genetically confirmed diagnosis of CPT II deficiency (four females and nine males) were included in the study (Table 1), with their ages at the time of analysis ranging 28–80 years (mean: 46.9 years). The clinical and molecular data of all patients have previously been reported in detail [16].

Fifty healthy individuals (27 females and 23 males) with no clinical or molecular evidence of mitochondrial disorder served as controls. The ages of the controls ranged 21–73 years (mean: 41 years).

The study was performed in accordance with the Helsinki Declaration. The study was approved by the ethics committee of the Medical Faculty of the Martin-Luther-University Halle-Wittenberg on 8 September 2012. Written informed consent was received from all patients. 

### 4.2. Measurement of FGF-21 Serum Concentration

FGF-21 serum concentration was measured in duplicate samples using ELISA (BioVendor, Brno, Czech Republic) according to the manufacturer’s instructions. A standard curve was created as described in the manual of the company BioVendor. The absolute concentration of FGF-21 in all samples was determined according to a linear standard curve.

The samples from patients were obtained during asymptomatic intervals. All samples were stored at –80 °C until analysis. The undetectable level was set as 0 pg/mL.

## Figures and Tables

**Table 1:** Epidemiological data of patients.

Patients	Gender	Age at Diagnosis (yrs.)	Age of First Attack (yrs.)	Attacks Per Year (*n*)	Intensity of Pain*	BMI	Genotype	FGF-21 (pg/mL)
1	F	19	6	5	3	23.9	p.Ser113Leu/p.Ser113Leu	47.0
2	F	34	6	1	4	25.9	p.Ser113Leu/p.Ser113Leu	66.5
3	M	33	8	1	4	26.6	p.Ser113Leu/p.Ser113Leu	152.5
4	M	53	8	7	7	27.1	p.Ser113Leu/p.Ser113Leu	82.0
5	M	21	10	7	4	24.2	p.Ser113Leu/p.Ser113Leu	57.0
6	M	54	5	1	7	24.2	p.Ser113Leu/c.1238delAG	37.0
7	M	25	4	85	4	30.7	p.Ser113Leu/c.340 + 1G > A	110.5
8	M	24	9	10	7	26.6	p.Ser113Leu/c.340 + 5G > A	25.5
9	M	45	10	1	4	30.0	p.Ser113Leu/c.182_203del22	56.5
10	M	22	10	1	4	26.4	p.Arg231Trp/p.Glu487Lys	12.0
11	M	57	17	11	5	24.9	p.Ser113Leu/p.Ser113Leu	0
12	F	53	18	50	4	27.9	p.Ser113Leu/p.Arg151Gln	57.5
13	F	39	15	6	5	20.4	p.Ser113Leu/p.Pro50His	57.0
Mean				8.8		26.3		66.2
Range (CI)				6.1–11.5		25.0–27.7		36.8–95.6
Controls (FGF-21)							Range (CI)	Mean
All (*n* = 50)							46.4–90.6	68.5
M (*n* = 23)							52.8–108.9	80.9
F (*n* = 27)							23.4–92.4	57.9

F: female, M: male; BMI: body mass index; *: intensity of pain on a visual analogue scale (VAS) (0: no pain at all, 10: unbearable severe pain) during an attack; CI: 95% confidence interval.
